# The evolved divergence of γ-secretase-susceptibility of homologous proteins Ngfrb and Nradd in zebrafish

**DOI:** 10.1186/s13104-021-05876-2

**Published:** 2021-12-20

**Authors:** Tanya Jayne, Morgan Newman, Lachlan Baer, Michael Lardelli

**Affiliations:** Department of Molecular and Biomedical Science, School of Biological Sciences, University of Adelaide, North Terrace, Adelaide, SA 5005 Australia

**Keywords:** Amyloid precursor protein secretases, Zebrafish, Gene duplication, NGFR protein

## Abstract

**Objective:**

NGFR/p75NTR and NRADD/NRH proteins are closely related structurally and are encoded by genes that arose from a duplication event early in vertebrate evolution. The transmembrane domain (TMD) of NGFR is cleaved by γ-secretase but there is conflicting data around the susceptibility to γ-secretase cleavage of NRADD proteins. If NGFR and NRADD show differential susceptibility to γ-secretase, then they can be used to dissect the structural constraints determining substrate susceptibility. We sought to test this differential susceptibility.

**Results:**

We developed labelled, lumenally-truncated forms of zebrafish Ngfrb and Nradd and a chimeric protein in which the TMD of Nradd was replaced with the TMD of Ngfrb. We expressed these in zebrafish embryos to test their susceptibility to γ-secretase cleavage by monitoring their stability using western immunoblotting. Inhibition of γ-secretase activity using DAPT increased the stability of only the Ngfrb construct. Our results support that only NGFR is cleaved by γ-secretase. Either NGFR evolved γ-secretase-susceptibility since its creation by gene duplication, or NRADD evolved to be refractory to γ-secretase. Protein structure outside of the TMD of NGFR is likely required for susceptibility to γ-secretase.

**Supplementary Information:**

The online version contains supplementary material available at 10.1186/s13104-021-05876-2.

## Objective/Introduction

The γ-secretase enzyme complex cleaves the TMD of type 1 membrane proteins. Usually this cleavage is dependent on removal of most of the lumenal domains of these proteins [1], although exceptions apparently exist [2]. Nearly 150 substrates of γ-secretase are known [3], but what differentiates γ-secretase substrates from type 1 membrane proteins not cleaved by this enzyme is unclear.

**Fig. 1:**
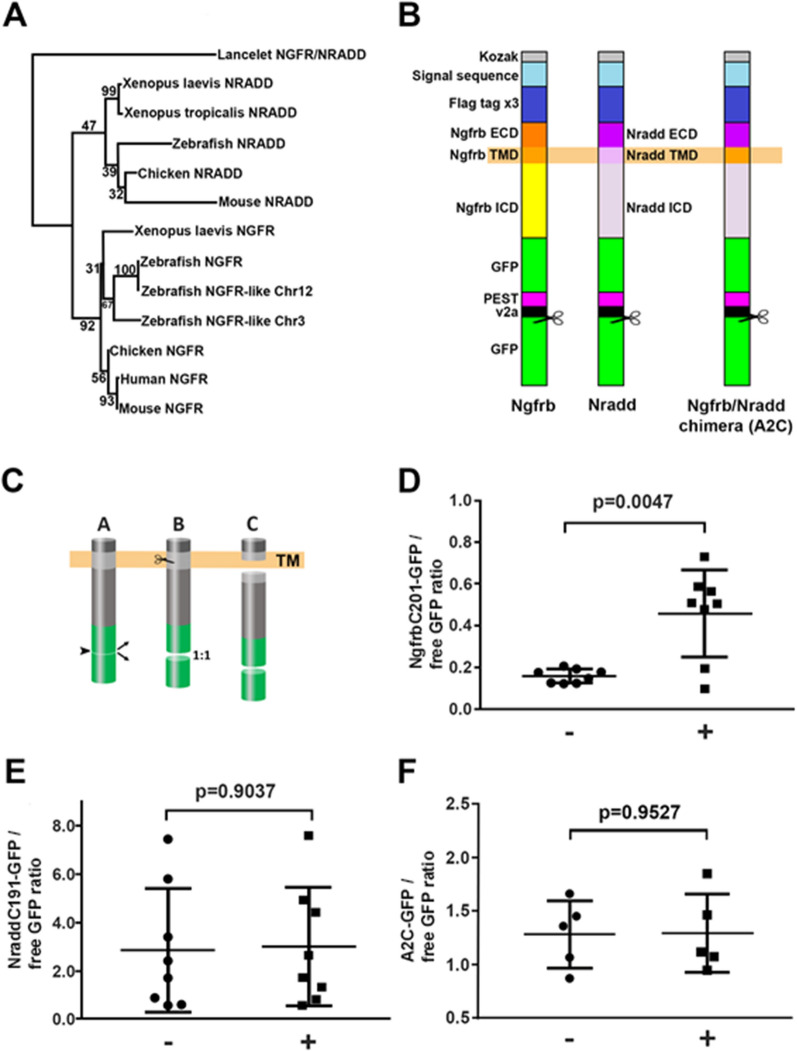
**A** Phylogenetic analysis of the relationships of chordate *NGFR* and *NRADD* genes. **B** The structure of the Ngfrb-, Nradd-, and Ngfrb/Nradd chimaeric-fusion proteins. Scissors indicate where the v2a sequence causes separation of the C-terminal free GFP from the remainder of each fusion protein. **C** During translation, the v2a sequence causes separation of the C-terminal free GFP from the remainder of each fusion protein so that the two protein fragments are initially present in a 1:1 ratio. The free GFP is then used as an internal control to assess the relative abundance of the N-terminal fusion protein fragment. **D**–**F** Comparison of the relative abundance of the N-terminal fragment of the Ngfrb- (**D**), Nradd- (**E**), and Ngfrb/Nradd chimaeric (**F**) -fusion proteins without (−) and in the presence of (+) γ-secretase DAPT. P-values are derived from two-tailed t-tests with Welch’s correction

The low-affinity nerve growth factor receptor (NGFR) is cleaved by γ-secretase [4, 5] and plays an important role in regulating cellular responses to NGF [5] and hypoxia [6]. Paralogous genes that arose during vertebrate evolution, the *death domain-containing membrane protein NRADD* (*NRADD*) family, also known as *Neurotrophic Receptor Homologue 1* and *2* (*NRH1*, *NRH2*), show closest sequence similarity to *NGFR*. A phylogenetic analysis including the basal chordate, the lancelet, suggests the possibility that the *NRADD* genes are more closely related structurally to the ancestral gene that duplicated to form *NGFR*, than is *NGFR* (Fig. 1A, Additional file 1). A deletion event in the *NRADD* evolutionary lineage means that the mammalian *NRADD* gene has lost sequence coding for N-terminal protein structures that is retained in the non-mammalian *NRADD* genes, e.g. *NRH1* [4]. In humans, *NRADD* has become a pseudogene, *neurotrophin receptor associated death domain, pseudogene*, *NRADDP*.

A 2003 study by Kanning et al. [4] found that neither *Xenopus laevis* NRADD (NRH1) protein nor mouse NRADD are cleaved by γ-secretase, implying either that NRADD proteins lost this susceptibility, or that NGFR gained it, after their divergence from a common ancestor. However, a 2004 study by Gowrishankar et al. [7] showed that mouse NRADD was, apparently, susceptible to γ-secretase cleavage if lumenal sequence was removed.

If NGFR and NRADD do, in fact, represent γ-secretase-susceptible and γ-secretase-refractory proteins respectively, then comparison of, and swapping of sequences between, these proteins might illuminate the structural determinants of γ-secretase susceptibility. However, the longer NRH1 form of NRADD is not found in mammals while the pseudotetraploid genome of *Xenopus laevis* complicates genetic analysis. Therefore, we sought to investigate the γ-secretase susceptibility of NGFR and NRADD in the versatile model organism, the zebrafish, *Danio rerio*.

## Main text

### Materials and methods

Phylogenetic analysis, DNA constructs, embryo injection and western immunoblotting, quantification, and statistical procedures are described in Additional files 1, 2 and 3.

### Results and discussion

An ancient whole-genome duplication (followed by loss of many, but not all, of the gene duplicates) in the teleost lineage, after its divergence from the tetrapods, means that a large minority (~ 26%) of the zebrafish orthologues of human genes exist as ohnologous pairs [8]. The ohnologues of human *NGFR* are *ngfra* and *ngfrb*. Zebrafish possess a single NRH1-like *NRADD* gene denoted *nradd*. A phylogenetic analysis using both Bayesian and Maximum likelihood methods confirmed these relationships (Fig. 1A and Additional files 1 and 2). Since the coding sequence of *ngfrb* had been determined from cDNA rather than predicted computationally from genomic DNA sequence (Additional file 1), we selected *ngfrb* to compare to zebrafish *nradd*.

After γ-secretase cleavage, the released cytosolic, C-terminal signalling domains of γ-secretase-susceptible proteins tend to be very unstable and can be difficult to monitor by western immunoblotting. However, rates of γ-secretase cleavage can be assessed by monitoring levels of the uncleaved (but N-terminally truncated) substrates. We have previously used this strategy to monitor γ-secretase activity in zebrafish embryos expressing mRNA coding for a derivative of the Amyloid Precursor Protein [9]. Injection of mRNAs encoding assay substrates into zebrafish zygotes is advantageous in that the large and uniform volume of these cells facilitates assay substrate expression at replicable levels. Also, subsequent interpretation of results is less complicated by questions regarding whether in vitro assay observations reflect the reality of protein processing in vivo, or whether any effects seen reflect events in “normal” cell types, (i.e. assay results might be distorted by the stress of cell culture conditions, or an abnormal/immortal genetic state of the cells etc.). Therefore, we designed three assay substrate expression constructs as described in Fig. 1B. (Coding sequences and additional cloning information for these constructs are given in Additional files 2 and 3). In each construct, sequence coding for an optimised secretory signal sequence is followed by three tandemly repeated FLAG tags. (Note, the presence of the FLAG tags was not exploited in our analysis described here). These FLAG tags are then followed by 15 codons from the extracellular domains of Ngfrb or Nradd. Directly after the 15 codons from Ngfrb or Nradd are the TMD and intracellular domain (ICD) fused to sequence coding for enhanced Green Fluorescent Protein (GFP), a PEST protein destabilisation sequence, and then a v2a sequence to separate, during translation, this upstream protein structure from an additional downstream, free enhanced GFP molecule that acts as an internal expression standard (see Fig. 1B, C) for western immunoblotting. Consequently, expression results are given as ratios of western immunoblot band intensities of potential substrate-GFP fusions (NgfrbC201-GFP and NraddC191-GFP) relative to their free GFP bands and these ratios are comparable between independent western immunoblots. In one of the three constructs, the TMD of supposedly γ-secretase-refractory Nradd was replaced with the γ-secretase-susceptible TMD of Ngfrb to give a “Ngfrb/Nradd chimera” (A2C-GFP). All three coding sequences were ligated into the Tol2 transposon-based expression construct pT2AL [10]. This allows mosaic, somatic expression of the protein constructs when injected, together with mRNA encoding Tol2 transposase, into fertilised zebrafish eggs at the 1-cell stage.

To observe the expression of the protein constructs and whether or not these are susceptible to γ-secretase cleavage, the expression vectors and transposase mRNA were injected into clutches of fertilised zebrafish eggs in embryo medium [11] which were then allowed to develop at 28.5 °C for 4 h. Each injected clutch was then divided into two groups and the γ-secretase inhibitor DAPT (dissolved in DMSO) added to one of these at a final concentration of 100 μM. (Both halves of the clutch also received DMSO to a final concentration of 1% v/v). After 24 h of development, yolks were removed from the embryos and the embryos then lysed in a sample buffer for western immunoblotting. Probing of these blots with an antibody recognising GFP allowed determination of the ratio of the abundance of Ngfrb-, Nradd-, or Ngfrb/Nradd chimera-proteins fused with GFP (NgfrbC201-GFP, NraddC191-GFP, or A2C-GFP) relative to free GFP. The lower this ratio, the more unstable (subject to γ-secretase cleavage) is the membrane-spanning substrate.

The results from these analyses of the Ngfrb, Nradd, or the Ngfrb/Nradd chimaera (A2C) fusion proteins are shown in Fig. 1D–F respectively (see Additional files 4 and 5 for raw data and analysis). The constructs proved quite toxic to the zebrafish embryos, probably due to the forced expression of the “death domains” in the ICDs of Ngfrb and Nradd. This likely contributed to variability in expression of the fusion proteins. However, the presence of the γ-secretase-inhibitor DAPT caused considerable stabilisation of the Ngfrb fusion protein, consistent with the susceptibility of Ngfrb to cleavage by γ-secretase (p = 0.0047). (The relatively lesser variability seen for the Ngfrb assay in the absence of the γ-secretase-inhibitor DAPT is expected since cleavage of the NgfrbC201-GFP fusion protein reduces its abundance relative to free GFP in the assay). γ-Secretase inhibition was not seen to increase the stability of the Nradd (p = 0.9037) or the Ngfrb/Nradd chimera (p = 0.9527) fusion proteins. These results support that Nradd (NRH1) is not a substrate of γ-secretase and that comparison of zebrafish Ngfrb and Nradd proteins can be performed to dissect the structural determinants of γ-secretase susceptibility. Interestingly, the inability of γ-secretase to cleave the Ngfrb/Nradd chimera fusion protein supports that structural information outside of the Ngfrb TMD is required for γ-secretase susceptibility. While it is possible that there are other factors (such as the artificial structure of the chimera) affecting the accessibility or cleavage rate of the γ-secretase recognition sequence indirectly, the conclusion we have drawn is consistent with previous observations comparing the γ-secretase substrate vasorin with the γ-secretase-refractory protein Itgβ1 [12].

## Limitations

A limitation of this study is that we have not attempted to identify and monitor the production of the unstable γ-secretase-cleavage products of Ngfrb and, potentially, Nradd by use of protease inhibitors. The experiments described above were already challenging as they were performed in developing embryos rather than in cultured cells. The injected expression constructs were quite toxic to development meaning that there was considerable death of embryos before they could be collected for lysis and western immunoblotting. Protease inhibition would have exacerbated this difficulty.

## Supplementary Information

**Additional file 1.** Phylogenetic analysis. The analysis used to generate the phylogenetic tree shown in Fig. 1A.

**Additional file 2.** Materials and methods. Materials and methods detailed description.

**Additional file 3.** Construct overviews and full sequences. Descriptions of construct structures.

**Additional file 4.** Supporting tables for western blot analyses. Densitometry data from western blots and calculated ratios.

**Additional file 5.** Raw western immunoblot data. Images of western blots from which densitometry data were derived.

## Data Availability

All data generated or analysed during this study are included in this published article and its Additional files.

## Abbreviations

DAPT: (2S)-*N*-[(3,5-Difluorophenyl)acetyl]-l-alanyl-2-phenyl]glycine 1,1-dimethylethyl ester
GFP: Green fluorescent protein
*NGFR*: Nerve growth factor receptor
*ngfrb*/Ngfrb: Zebrafish nerve growth factor receptor gene/protein
*nradd*/Nradd: Zebrafish neurotrophin receptor associated death domain gene/protein
*NRADDP*: Neurotrophin receptor associated death domain, pseudogene
*NRH1*: Neurotrophin receptor homolog 1
*p75NTR*: P75 neurotrophin receptor
PEST: Proline (P), glutamic acid (E), serine (S), and threonine (T) rich sequence motif determining protein stability
TMD: Transmembrane domain
v2a: Viral 2a sequence
v/v: Millilitres per 100 mL total volume in water
