# Supplementary material for: The evolved divergence of γ-secretase-susceptibility of homologous proteins Ngfrb and Nradd in zebrafish
Source: BMC Res Notes. 2021 Dec 20;14:460. doi: 10.1186/s13104-021-05876-2 (PMC8686249; doi:10.1186/s13104-021-05876-2)
Supplement: Supplementary file 1 — Additional file 1. Phylogenetic analysis. The analysis used to generate the phylogenetic tree shown in Fig. 1A. [file 13104_2021_5876_MOESM1_ESM.docx]

**Additional File 1. Phylogenetic analysis**

**The analysis used to generate the phylogenetic tree shown in Figure 1A**

To investigate NGFR (p75NTR) and NRADD (NRH1) cleavage *in vivo* using zebrafish, we first validated the orthology of these genes. A tblastn search performed against the zebrafish genome using the entire putative protein sequence of human *NGFR*, constrained to “RefSeq_RNA”, returned candidate orthologues of the human *NGFR* gene on zebrafish chromosomes 3, 12 and 16. At the time of the original analysis, chromosome 12 appeared to hold two almost identical copies of the gene at different loci, which we suspected was due to a recent duplication event. The position of the duplicate appears to have been revised to chromosome 3 in the latest genome build (GRCz11). The top tblastn hit, *nerve growth factor receptor b* (*ngfrb*), on chromosome 12, has the greatest query coverage (percentage of the sequence aligned to a sequence in GenBank) to human *NGFR* (93%), so we tentatively referred to this as “zebrafish Ngfr”. A tblastn search performed against the zebrafish genome using *NRADD* from *Xenopus laevis* returned the computer predicted sequence for *neurotrophin receptor associated death domain* (*nradd*) on chromosome 16 with 100% query coverage to Xenopus *NRADD*. The only other strong zebrafish *Nradd* candidate returned was *ngfrb*, which we had already established most likely represents *Ngfr* in zebrafish. Therefore, we predict that *nradd* is most likely a *Nradd*-orthologous gene in zebrafish.

Phylogenetic analysis was conducted using R v4.1.1 (1). NCBI protein accessions listed in Table 1 below were used to download amino acid sequences with the *rentrez* package (2). Protein sequences were aligned using the Clustal Omega algorithm (3) implemented by the *msa* package (4). *Gblocks* software (5) was then run from the command line on the resulting multiple sequence alignment to eliminate poorly aligned and divergent positions (Figures 2 and 3 below), with parameters set for less stringent selection as recommended for short alignments (Minimum Number Of Sequences For A Conserved Position: 7, Minimum Number Of Sequences For A Flanking Position: 8, Maximum Number Of Contiguous Nonconserved Positions: 10, Minimum Length Of A Block: 5, Allowed Gap Positions: None, Use Similarity Matrices: Yes). Phylogenetic tree estimation was performed using maximum likelihood methods with the JTT model implemented by the *phangorn* package (6).  *Branchiostoma floridae* (lancelet) was used as an out-group as this was the most distant relative to zebrafish that returned a result when conducting tblastn searches using human *NGFR* and *X. laevis* *NRADD*. Interestingly, tblastn searches of the lancelet genome using both *NGFR* and *NRADD* returned the same gene in lancelet. As the chicken genome contains both *NGFR* and *NRADD*-like sequences, tblastn searches of the lancelet genome using both full-length chicken sequences were performed to confirm the preliminary findings. These searches returned results identical to those using human *NGFR* and *X. laevis* *NRADD*. This supports that there is only a single *NGFR*- and *NRADD*-like gene in this basal chordate and that *NGFR* and *NRADD* arose from a gene duplication event early in vertebrate evolution.

A dendrogram modelling the phylogenetic relationships of NGFR and its homologs demonstrated NGFR proteins clustering together and NRADD proteins clustering together in separate clades (Figure 1.A in the main text). This supports that the sequence *nradd* on chromosome 16 of zebrafish is indeed the orthologue of *Nradd*.

| **Table 1. NCBI accession numbers of sequences used in phylogenetic analyses** | | | |
| --- | --- | --- | --- |
| **Species and NGFR/NRADD?** | **NCBI name** | **NCBI Accession nucleotide** | **NCBI Accession protein** |
| Zebrafish NGFR | Ngfrb | NM_001198660.1 | NP_001185589.1 |
| Zebrafish NGFR like 12 | Ngfrb (duplicate) | XM_003199576.1 | XP_003199624.1 |
| Zebrafish NGFR like 3 | Ngfra | XM_003198085.3 | XP_003198133.1 |
| Zebrafish NRADD | Ngfr | XM_695893.4 | XP_700985.3 |
| Xenopus tropicalis NRADD | Nradd | NM_001007998.2 | NP_001007999.2 |
| Xenopus L NGFR | Ngfr L | NM_001088466.1 | NP_001081935.1 |
| Xenopus laevis NRADD | Nradd S | NM_001091773.1 | NP_001085242.1 |
| Mouse NGFR | NGFR | NM_033217.3 | NP_150086.2 |
| Mouse NRADD | NRADD | NM_026012.2 | NP_080288.1 |
| Chicken NGFR | NGFR | NM_001146133.1 | NP_001139605.1 |
| Chicken NRADD | TNFR 16-like | XM_418509.3 | XP_418509.3 |
| Human NGFR | TNFR superfamily 16 | NM_002507.3 | p08138 |
| Human NRADD | NRADDP | NR_024046 | N/A pseudogene |
| Lancelet NGFR/NRADD | *Branchiostoma floridae* hypothetical protein | XM_002588230.1 | XP_002588276.1 |


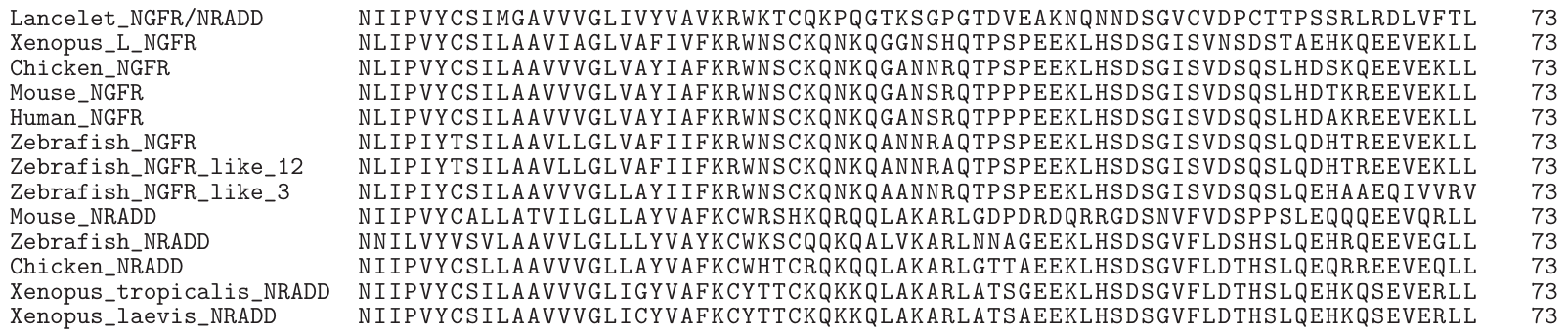


**Figure 2. Aligned protein sequences used in phylogenetic analyses.** Figure 3 below shows how these sequences were derived.


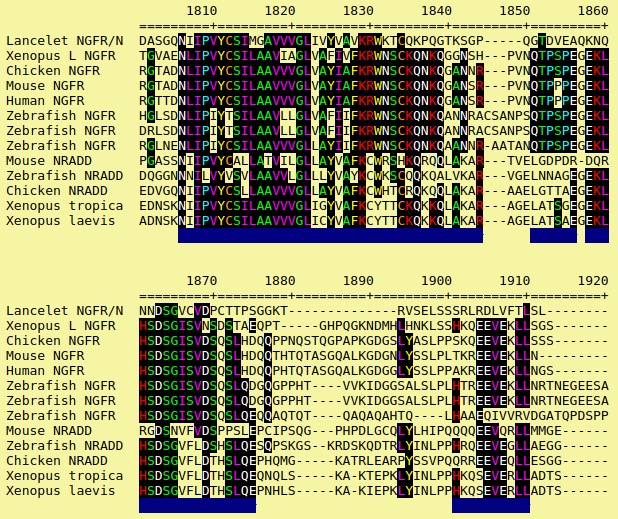


**Figure 3. The derivation of the protein sequences shown in Figure 2.** Amino acid positions passing the *Gblocks* selection parameters described above are indicated below the sequences with a blue bar. Positions with at least 7 conserved amino acids across all sequences are highlighted in black.

**References**

1. R Core Team (2021). R: A language and environment for statistical computing. R Foundation for Statistical Computing, Vienna, Austria. URL https://www.R-project.org/.
2. Winter DJ (2017). “rentrez: an R package for the NCBI eUtils API.” The R Journal, 9, 520–526.
3. Sievers F, Wilm A, Dineen D, et al. Fast, scalable generation of high-quality protein multiple sequence alignments using Clustal Omega. Mol Syst Biol. 2011;7:539. Published 2011 Oct 11. doi:10.1038/msb.2011.75
4. Bodenhofer U, Bonatesta E, Horejs-Kainrath C, Hochreiter S (2015). “msa: an R package for multiple sequence alignment.” Bioinformatics, 31(24), 3997–3999. doi: 10.1093/bioinformatics/btv494.
5. Gerard Talavera, Jose Castresana, Improvement of Phylogenies after Removing Divergent and Ambiguously Aligned Blocks from Protein Sequence Alignments, Systematic Biology, Volume 56, Issue 4, August 2007, Pages 564–577, https://doi.org/10.1080/10635150701472164
6. Schliep K (2011). “phangorn: phylogenetic analysis in R.” Bioinformatics, 27(4), 592–593. doi: 10.1093/bioinformatics/btq706.
