# Supplementary material for: The evolved divergence of γ-secretase-susceptibility of homologous proteins Ngfrb and Nradd in zebrafish
Source: BMC Res Notes. 2021 Dec 20;14:460. doi: 10.1186/s13104-021-05876-2 (PMC8686249; doi:10.1186/s13104-021-05876-2)
Supplement: Supplementary file 2 — Additional file 2. Materials and methods. Materials and methods detailed description. [file 13104_2021_5876_MOESM2_ESM.docx]

**Additional File 2. Materials and Methods**

**Gene orthologue identification**

Alignments and tree building were conducted using the Geneious software suite, version 5.6.7 (http://www.geneious.com, [12]). Alignments were performed with the following constraints: Cost matrix: Identity, Gap open penalty: 10, Gap extension penalty: 3, Alignment: Global. Bayesian trees were produced using the “Mr Bayes” program with the following constraints: Substitution model: GTR, Outgroup: Lancelet NGFR, and the rest as default. (Discussed in greater detail in Additional File 1).

**Constructs**

The NgfrbC201-GFP, NraddC191-GFP and A2C-GFP (Ngfrb/Nradd chimera) constructs used in these analyses were assembled from synthetic DNA produced by Biomatik (complete DNA sequences are provided in Additional File 3). These DNA sequences in the pBMH vector (provided by Biomatik) were digested using *Bam H*I and *Cla* I in independent reactions and ligated into pT2AL200R150G between the *Bam H*I and *Cla* I sites.

**DNA microinjection of zebrafish embryos and treatment with DAPT.**

Tol2 transposase plasmid (pCS-TP) was linearised using *Not* 1 (NEB) and mRNA was transcribed *in vitro* using the mMESSAGE mMACHINE SP6 Kit (Ambion Inc.). Fertilised embryos were injected with a solution containing 100ng/µl plasmid DNA and approximately 50ng/µl Tol2 transposase mRNA at the one cell stage. ~50 embryos injected with the injection solution above were placed into 35mm x 10mm petri dishes with 2 ml E3 medium (15mM NaCl, 0.5mM KCl, 1mM MgSO_4_, 0.15mM KH_2_PO_4_, 0.05mM Na_2_HPO_4_, 1mM CaCl_2_, 0.7mM NaHCO_3_). At 4 hours post fertilisation (hpf) embryos were treated with 100µM DAPT (In solutionTM γ-secretase inhibitor IX, Calbiochem, San Diego, CA, USA) in 1% DMSO in E3 medium. Embryos were maintained at 28°C in a humid incubator. At 24 hpf embryos were visualised under UV light for GFP expression. Embryos expressing GFP were selected for protein extraction.

**Western Immunoblot analyses**

Dechorioned and de-yolked embryos were lysed by placement in sample buffer (2% sodium dodecyl sulfate (SDS), 5% β-mercaptoethanol, 25% v/v glycerol, 0.0625 M Tris-HCl (pH 6.8), and bromophenol blue) followed immediately by heating to 95°C for 10 min, before storage at -80°C prior to protein separation on 4-12% SDS polyacrylamide gels. Proteins were transferred to nitrocellulose membrane in buffer (25mM Tris, 192mM glycine, 0.1% sodium lauryl sulfate, 20% methanol in MilliQ H2O) at 10V for 1hr. When immunoblotting, all membranes were blocked with 5% Western Blocking Reagent (Roche, Indianapolis, IN, USA) in TBST. GFP immunoblots were incubated in a 1/5,000 dilution of anti-GFP antibodies (Rockland Immunochemicals Inc., Gilbertsville, PA, USA) in TBST containing 0.5% Western Blocking Reagent. Subsequently, blots were washed in TBST and then incubated in a 1/10,000 dilution of donkey anti-Goat IgG (Rockland Immunochemicals Inc., Gilbertsville, PA, USA). Blot membranes were then washed three times for 10 minutes in TBST and antibody-bound proteins then visualised with luminol reagents (Amresco, Ohio, USA or Thermo Scientific, Rockford, USA) by the ChemiDocTM MP imaging system (Bio-Rad, Hercules, CA, USA). The NgfrbC201-GFP, NraddC191-GFP and A2C-GFP protein bands were visualised at ~61kDa, 57kDa and 57kDa respectively. Using Image Lab software (Bio-Rad), densitometry analyses were performed on the protein bands relevant for each sample and on the free GFP internal reference protein band of each sample. Densitometry values, and densitometry value ratios of NgfrbC201-GFP, NraddC191-GFP and A2C-GFP bands relative to their free GFP bands are given in Additional File 4.

**Statistical analyses**

Unpaired, two-tailed t-tests with Welch’s correction (assuming Gaussian distribution), were used to compare densitometry ratios from samples with and without γ-secretase inhibition using GraphPad Prism version 8.0.0 for Windows, GraphPad Software, San Diego, California USA, www.graphpad.com. P values of < 0.05 were considered to be significant. We did not remove any outliers during the analyses.
