# Supplementary material for: The evolved divergence of γ-secretase-susceptibility of homologous proteins Ngfrb and Nradd in zebrafish
Source: BMC Res Notes. 2021 Dec 20;14:460. doi: 10.1186/s13104-021-05876-2 (PMC8686249; doi:10.1186/s13104-021-05876-2)
Supplement: Supplementary file 3 — Additional file 3. Construct overviews and full sequences. Descriptions of construct structures. [file 13104_2021_5876_MOESM3_ESM.docx]

**Additional File 3. Construct overviews and full sequences**

1. **Sequence design for the NgfrbC201-GFP-v2a-GFP construct**

Different regions of the sequence are indicated with colours corresponding to the legend below:

- BamHI-Kozak-START-SS-FlagTag1-FlagTag2-FlagTag3-Ngrfb(CTF)-GFP-*BspE* I-MODCPEST-*BspE* I-v2a-*Xba* 1-GFP-*Xba* 1-STOP-*Not* I-*Cla* I
- Capitalised C in FlagTag2 = silent mutation, capitalised T in FlagTag3 = silent mutation

Sequence of NgfrbC201-GFP-v2a-GFP construct:

cgggatccgccaccatgtggtggcgcctgtggtggctgctgctgctgctgctgctgctgtggcccatggtgtgggccgattacaaggatgacgacgataaggattacaagga**C**gacgacgataaggattacaaggatgacga**T**gataagaccacgactgaccctgggcaaaagagactacatgggcttagcgacaacctcattcccatctacacctccatactggctgcggtgctgttgggcctggtggccttcatcatatttaaacggtggaacagttgtaagcagaataagcaagccaataaccgagcgtgcagcgcaaaccccagtcagactccttctcctgagggagagaaactccacagtgacagcggcatctcagtggacagtcagagcctgcaggacggccaggggcctccacatacagtggtcaagatagatggaggttcggctctgtctttgcccttacacacacgggaggaggtggagaaactgctaaatcgtaccaatgaaggagaggagtcagcagccaatgaggagaccgactggtgcagcctagccgggctacttggatacaaagaagaacacattgctaatttcaagcaggaggaacggcccatccaggcacttctgtcgcactgggcgagccaggattcggctaacattgatacgctttgcacagctctgaagaagatcaacagagaggatattgcgcaaagcatcattgtcaaaccaactgccacatctgccgtaatggtgagcaagggcgaggagctgttcaccggggtggtgcccatcctggtcgagctggacggcgacgtaaacggccacaagttcagcgtgtccggcgagggcgagggcgatgccacctacggcaagctgaccctgaagttcatctgcaccaccggcaagctgcccgtgccctggcccaccctcgtgaccaccctgacctacggcgtgcagtgcttcagccgctaccccgaccacatgaagcagcacgacttcttcaagtccgccatgcccgaaggctacgtccaggagcgcaccatcttcttcaaggacgacggcaactacaagacccgcgccgaggtgaagttcgagggcgacaccctggtgaaccgcatcgagctgaagggcatcgacttcaaggaggacggcaacatcctggggcacaagctggagtacaactacaacagccacaacgtctatatcatggccgacaagcagaagaacggcatcaaggtgaacttcaagatccgccacaacatcgaggacggcagcgtgcagctcgccgaccactaccagcagaacacccccatcggcgacggccccgtgctgctgcccgacaaccactacctgagcacccagtccgccctgagcaaagaccccaacgagaagcgcgatcacatggtcctgctggagttcgtgaccgccgccgggatcactctcggcatggacgagctgtacaagggctccggacatggcttcccgccggcggtggcggcgcaggatgatggcacgctgcccatgtcttgtgcccaggagagcgggatggaccgtcaccctgcagcctgtgcttctgctaggatcaatgtgggctccggagctacaaatttctctctgttgaaacaggctggtgacgtcgaggagaatcctggcccatctagaatggtgagcaagggagaggagctgttcacaggagtggtgcctatcctggtggagctggacggagacgtgaacggacacaagttcagcgtgagcggagagggagagggagacgctacatacggaaagctgacactgaagttcatctgtacaacaggaaagctgcctgtgccttggcctacactggtgacaacactgacatacggagtgcagtgtttcagcagataccctgaccacatgaagcagcacgacttcttcaagagcgctatgcctgagggatacgtgcaggagagaacaatcttcttcaaggacgacggaaactacaagacaagagctgaggtgaagttcgagggagacacactggtgaacagaatcgagctgaagggaatcgacttcaaggaggacggaaacatcctgggacacaagctggagtacaactacaacagccacaacgtgtacatcatggctgacaagcagaagaacggaatcaaggtgaacttcaagatcagacacaacatcgaggacggaagcgtgcagctggctgaccactaccagcagaacacacctatcggagacggacctgtgctgctgcctgacaaccactacctgagcacacagagcgctctgagcaaggaccctaacgagaagagagaccacatggtgctgctggagttcgtgacagctgctggaatcacactgggaatggacgagctgtacaagtctagataggcggccgcatcgatgg

1. **Sequence design for the NraddC191-GFP-v2a-GFP construct**

Different regions of the sequence are indicated with colours corresponding to the legend below:

- BamHI-Kozak-START-SS-FlagTag1-FlagTag2-FlagTag3-Nradd(CTF)-GFP-*BspE* I-MODCPEST-*BspE* I-v2a-*Xba* 1-GFP-*Xba* 1-STOP-*Not* I-*Cla* I
- Capitalised C in FlagTag2 = silent mutation, capitalised T in FlagTag3 = silent mutation

Sequence of NraddC191-GFP-v2a-GFP construct:

cgggatccgccaccatgtggtggcgcctgtggtggctgctgctgctgctgctgctgctgtggcccatggtgtgggccgattacaaggatgacgacgataaggattacaagga**C**gacgacgataaggattacaaggatgacga**T**gataagggctctggagcccctagactcacaccacaggaccaaggtggaaacaacaacatcttggtgtatgtgtctgttttggctgctgtggtgcttggcctgctgctctacgttgcctacaaatgctggaaatcatgtcagcagaagcaggctctggtgaaggcccgggtcggagagctgaataatgcaggggaaggagagaaactgcacagcgacagtggtgttttcctggactctcacagccttcaggaaagccagcctagcaaaggcagtaaacgggacagcaaacaggatacacggctctacataaacctgcctccgcacagacaggaggaggtagagggacttctggctgaggggggcaatcgaagctggaaacagctagccgctacactaggctacgaacaggagcgcgtggacgtctttggacggggccaggaccccatccacaccctcatgaccgattggtcccagcaggaaggctccacattgggtttgttgtgttcagctctgactcgcatcgagcgaccagacatcatcactgctctgaccgccccaacgcaaggagtatcagtggtcatggtgagcaagggcgaggagctgttcaccggggtggtgcccatcctggtcgagctggacggcgacgtaaacggccacaagttcagcgtgtccggcgagggcgagggcgatgccacctacggcaagctgaccctgaagttcatctgcaccaccggcaagctgcccgtgccctggcccaccctcgtgaccaccctgacctacggcgtgcagtgcttcagccgctaccccgaccacatgaagcagcacgacttcttcaagtccgccatgcccgaaggctacgtccaggagcgcaccatcttcttcaaggacgacggcaactacaagacccgcgccgaggtgaagttcgagggcgacaccctggtgaaccgcatcgagctgaagggcatcgacttcaaggaggacggcaacatcctggggcacaagctggagtacaactacaacagccacaacgtctatatcatggccgacaagcagaagaacggcatcaaggtgaacttcaagatccgccacaacatcgaggacggcagcgtgcagctcgccgaccactaccagcagaacacccccatcggcgacggccccgtgctgctgcccgacaaccactacctgagcacccagtccgccctgagcaaagaccccaacgagaagcgcgatcacatggtcctgctggagttcgtgaccgccgccgggatcactctcggcatggacgagctgtacaagggctccggacatggcttcccgccggcggtggcggcgcaggatgatggcacgctgcccatgtcttgtgcccaggagagcgggatggaccgtcaccctgcagcctgtgcttctgctaggatcaatgtgggctccggagctacaaatttctctctgttgaaacaggctggtgacgtcgaggagaatcctggcccatctagaatggtgagcaagggagaggagctgttcacaggagtggtgcctatcctggtggagctggacggagacgtgaacggacacaagttcagcgtgagcggagagggagagggagacgctacatacggaaagctgacactgaagttcatctgtacaacaggaaagctgcctgtgccttggcctacactggtgacaacactgacatacggagtgcagtgtttcagcagataccctgaccacatgaagcagcacgacttcttcaagagcgctatgcctgagggatacgtgcaggagagaacaatcttcttcaaggacgacggaaactacaagacaagagctgaggtgaagttcgagggagacacactggtgaacagaatcgagctgaagggaatcgacttcaaggaggacggaaacatcctgggacacaagctggagtacaactacaacagccacaacgtgtacatcatggctgacaagcagaagaacggaatcaaggtgaacttcaagatcagacacaacatcgaggacggaagcgtgcagctggctgaccactaccagcagaacacacctatcggagacggacctgtgctgctgcctgacaaccactacctgagcacacagagcgctctgagcaaggaccctaacgagaagagagaccacatggtgctgctggagttcgtgacagctgctggaatcacactgggaatggacgagctgtacaagtctagataggcggccgcatcgatgg

1. **Sequence design for the A2C-GFP-v2a-GFP construct**

Different regions of the sequence are indicated with colours corresponding to the legend below:

- BamHI-Kozak-START-SS-FlagTag1-FlagTag2-FlagTag3- Nradd ECD- Ngfrb TMD- Nradd ICD-GFP-*BspE* I-MODCPEST-*BspE* I-v2a-*Xba* 1-GFP-*Xba* 1-STOP-*Not* I-*Cla* I
- Capitalised C in FlagTag2 = silent mutation, capitalised T in FlagTag3 = silent mutation

Sequence of A2C-GFP-v2a-GFP construct:

5’-cgggatccgccaccatgtggtggcgcctgtggtggctgctgctgctgctgctgctgctgtggcccatggtgtgggccgattacaaggatgacgacgataaggattacaagga**C**gacgacgataaggattacaaggatgacga**T**gataagggctctggagcccctagactcacaccacaggaccaaggtggaaacaacctcattcccatctacacctccatactggctgcggtgctgttgggcctggtggccttcatcatatttaaatgctggaaatcatgtcagcagaagcaggctctggtgaaggcccgggtcggagagctgaataatgcaggggaaggagagaaactgcacagcgacagtggtgttttcctggactctcacagccttcaggaaagccagcctagcaaaggcagtaaacgggacagcaaacaggatacacggctctacataaacctgcctccgcacagacaggaggaggtagagggacttctggctgaggggggcaatcgaagctggaaacagctagccgctacactaggctacgaacaggagcgcgtggacgtctttggacggggccaggaccccatccacaccctcatgaccgattggtcccagcaggaaggctccacattgggtttgttgtgttcagctctgactcgcatcgagcgaccagacatcatcactgctctgaccgccccaacgcaaggagtatcagtggtcatggtgagcaagggcgaggagctgttcaccggggtggtgcccatcctggtcgagctggacggcgacgtaaacggccacaagttcagcgtgtccggcgagggcgagggcgatgccacctacggcaagctgaccctgaagttcatctgcaccaccggcaagctgcccgtgccctggcccaccctcgtgaccaccctgacctacggcgtgcagtgcttcagccgctaccccgaccacatgaagcagcacgacttcttcaagtccgccatgcccgaaggctacgtccaggagcgcaccatcttcttcaaggacgacggcaactacaagacccgcgccgaggtgaagttcgagggcgacaccctggtgaaccgcatcgagctgaagggcatcgacttcaaggaggacggcaacatcctggggcacaagctggagtacaactacaacagccacaacgtctatatcatggccgacaagcagaagaacggcatcaaggtgaacttcaagatccgccacaacatcgaggacggcagcgtgcagctcgccgaccactaccagcagaacacccccatcggcgacggccccgtgctgctgcccgacaaccactacctgagcacccagtccgccctgagcaaagaccccaacgagaagcgcgatcacatggtcctgctggagttcgtgaccgccgccgggatcactctcggcatggacgagctgtacaagggctccggacatggcttcccgccggcggtggcggcgcaggatgatggcacgctgcccatgtcttgtgcccaggagagcgggatggaccgtcaccctgcagcctgtgcttctgctaggatcaatgtgggctccggagctacaaatttctctctgttgaaacaggctggtgacgtcgaggagaatcctggcccatctagaatggtgagcaagggagaggagctgttcacaggagtggtgcctatcctggtggagctggacggagacgtgaacggacacaagttcagcgtgagcggagagggagagggagacgctacatacggaaagctgacactgaagttcatctgtacaacaggaaagctgcctgtgccttggcctacactggtgacaacactgacatacggagtgcagtgtttcagcagataccctgaccacatgaagcagcacgacttcttcaagagcgctatgcctgagggatacgtgcaggagagaacaatcttcttcaaggacgacggaaactacaagacaagagctgaggtgaagttcgagggagacacactggtgaacagaatcgagctgaagggaatcgacttcaaggaggacggaaacatcctgggacacaagctggagtacaactacaacagccacaacgtgtacatcatggctgacaagcagaagaacggaatcaaggtgaacttcaagatcagacacaacatcgaggacggaagcgtgcagctggctgaccactaccagcagaacacacctatcggagacggacctgtgctgctgcctgacaaccactacctgagcacacagagcgctctgagcaaggaccctaacgagaagagagaccacatggtgctgctggagttcgtgacagctgctggaatcacactgggaatggacgagctgtacaagtctagataggcggccgcatcgatgg-3’
