# Supplementary material for: The evolved divergence of γ-secretase-susceptibility of homologous proteins Ngfrb and Nradd in zebrafish
Source: BMC Res Notes. 2021 Dec 20;14:460. doi: 10.1186/s13104-021-05876-2 (PMC8686249; doi:10.1186/s13104-021-05876-2)
Supplement: Supplementary file 4 — Additional file 4. Supporting tables for western blot analyses. Densitometry data from western blots and calculated ratios. [file 13104_2021_5876_MOESM4_ESM.docx]

**Additional File 4. Supporting tables for western blot analyses**

**Table 1. Western immunoblot band intensities and intensity ratios for Figure 1.D and 1.E**

| **NgfrbC201-GFP injected, without DAPT treatment and collected at 24hpf** | | |
| --- | --- | --- |
| **NgfrbC201-GFP** | **free GFP** | **NgfrbC201-GFP/free GFP** |
| 4099260 | 21408354 | 0.191479457 |
| 2265235 | 18651920 | 0.121447819 |
| 2760660 | 13464144 | 0.205037914 |
| 4012008 | 31961958 | 0.125524475 |
| 3141090 | 24936450 | 0.1259638 |
| 1371942 | 7807518 | 0.175720632 |
| 1161584 | 6491984 | 0.178925888 |
| 784560 | 5346448 | 0.146744156 |
| **NgfrbC201-GFP injected, with DAPT treatment and collected at 24hpf** | | |
| **NgfrbC201-GFP** | **free GFP** | **NgfrbC201-GFP/free GFP** |
| 9307074 | 19496964 | 0.477360167 |
| 11522280 | 15773135 | 0.73050031 |
| 9746802 | 19327971 | 0.504284801 |
| 2743486 | 27871788 | 0.098432365 |
| 8096400 | 15898455 | 0.509257032 |
| 3262120 | 5782000 | 0.564185403 |
| 1073216 | 5489744 | 0.195494726 |
| 3258768 | 5566128 | 0.585464078 |
|  |  |  |
| **NraddC191-GFP injected, without DAPT treatment and collected at 24hpf** | | |
| **NraddC191-GFP** | **free GFP** | **NraddC191-GFP/free GFP** |
| 1491240 | 259845 | 5.738959764 |
| 3290364 | 445676 | 7.382861092 |
| 3774136 | 1123326 | 3.359786918 |
| 14486528 | 27954624 | 0.518215806 |
| 9071304 | 16119450 | 0.562755181 |
| 2069262 | 2441270 | 0.847617019 |
| 7176832 | 3022704 | 2.374308566 |
| 4717200 | 2825728 | 1.669375113 |
| **NraddC191-GFP injected, with DAPT treatment and collected at 24hpf** | | |
| **NraddC191-GFP** | **free GFP** | **NraddC191-GFP/free GFP** |
| 2157554 | 286594 | 7.528259489 |
| 2754024 | 563472 | 4.887596899 |
| 1687808 | 386528 | 4.366586638 |
| 16519654 | 30580320 | 0.5402054 |
| 15641976 | 20213136 | 0.773852014 |
| 6142512 | 3632768 | 1.690862725 |
| 4824704 | 3712496 | 1.299584969 |
| 6105872 | 2351728 | 2.59633427 |

**Table 2. Western immunoblot band intensities and intensity ratios for Figure 1.F**

| **A2C-GFP injected without DAPT treatment and collected at 24hpf** | | |
| --- | --- | --- |
| **A2C-GFP** | **free GFP** | **A2C-GFP/free GFP** |
| 5968380 | 4111428 | 1.451656213 |
| 3976478 | 2930257 | 1.357040696 |
| 3134032 | 1885752 | 1.661953428 |
| 2948385 | 2764267 | 1.066606446 |
| 5134025 | 5889052 | 0.871791419 |
| **A2C-GFP injected with DAPT treatment and collected at 24hpf** | | |
| **A2C-GFP** | **free GFP** | **A2C-GFP/free GFP** |
| 4955500 | 2678104 | 1.850376236 |
| 2455871 | 1668183 | 1.472183208 |
| 3267396 | 3427666 | 0.953242235 |
| 3248465 | 2896872 | 1.121369878 |
| 4519932 | 4193688 | 1.077794056 |
