# Supplementary material for: The evolved divergence of γ-secretase-susceptibility of homologous proteins Ngfrb and Nradd in zebrafish
Source: BMC Res Notes. 2021 Dec 20;14:460. doi: 10.1186/s13104-021-05876-2 (PMC8686249; doi:10.1186/s13104-021-05876-2)
Supplement: Supplementary file 5 — Additional file 5. Raw western immunoblot data. Images of western blots from which densitometry data were derived. [file 13104_2021_5876_MOESM5_ESM.docx]

**Additional File 5. Raw western immunoblot data**

Topmost arrows on all figures presented below represent bands from which intensity values were taken for NgfrbC201-GFP, NraddC191-GFP and A2C-GFP columns in tables in Additional File 4, while bottom arrows represent bands from which intensity values were taken for free GFP.


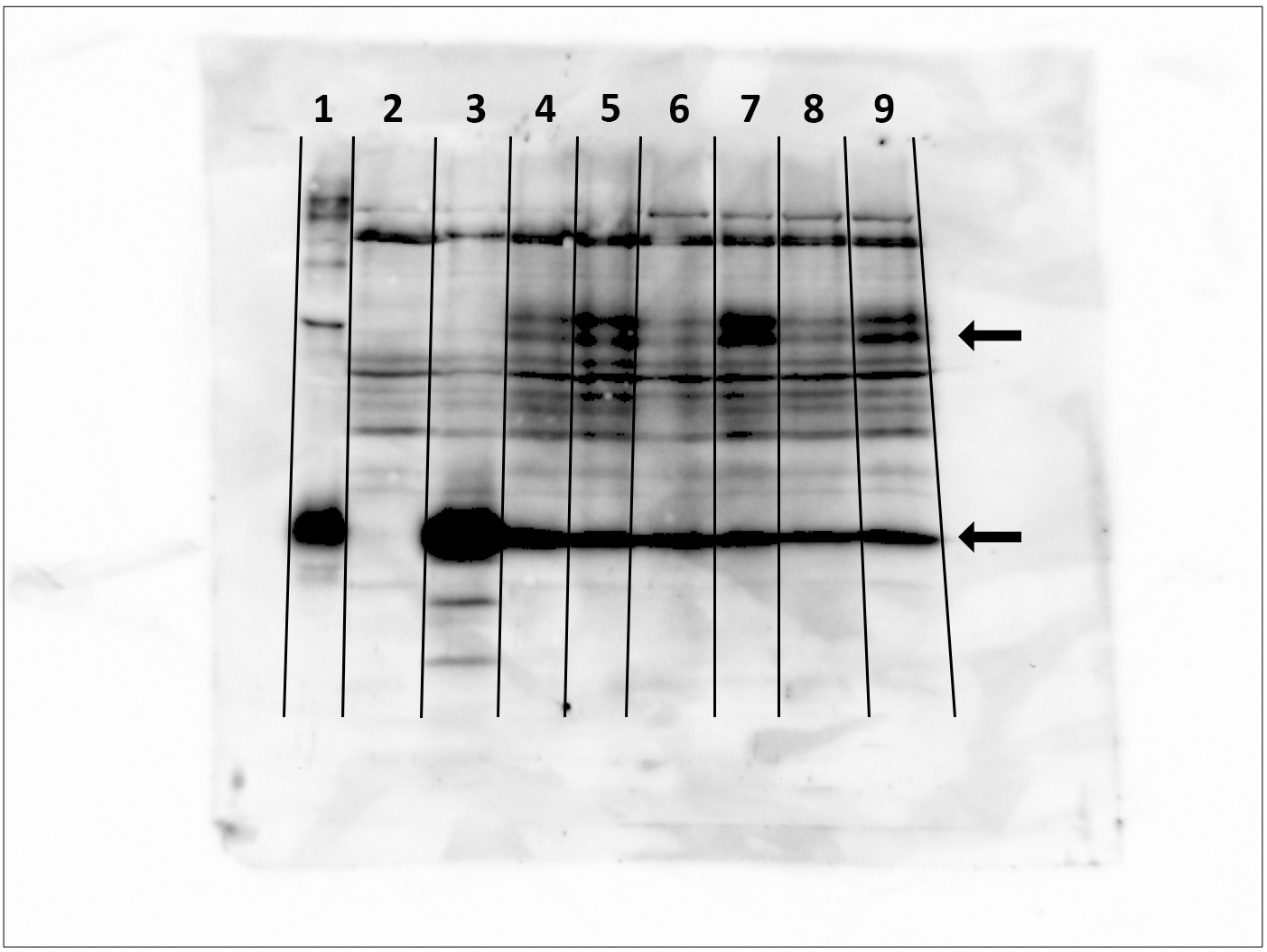


**Figure 5.1. Ngfrb western immunoblot 1** - 1) Marker, 2) uninjected, 3) pT2AL200R150G (Tol2) Empty Vector injection, 4) NgfrbC201-GFP sample group 1 untreated, 5) NgfrbC201-GFP sample group 1 DAPT treated, 6) NgfrbC201-GFP sample group 2 untreated, 7) NgfrbC201-GFP sample group 2 DAPT treated, 8) NgfrbC201-GFP sample group 3 untreated, 9) NgfrbC201-GFP sample group 3 DAPT treated


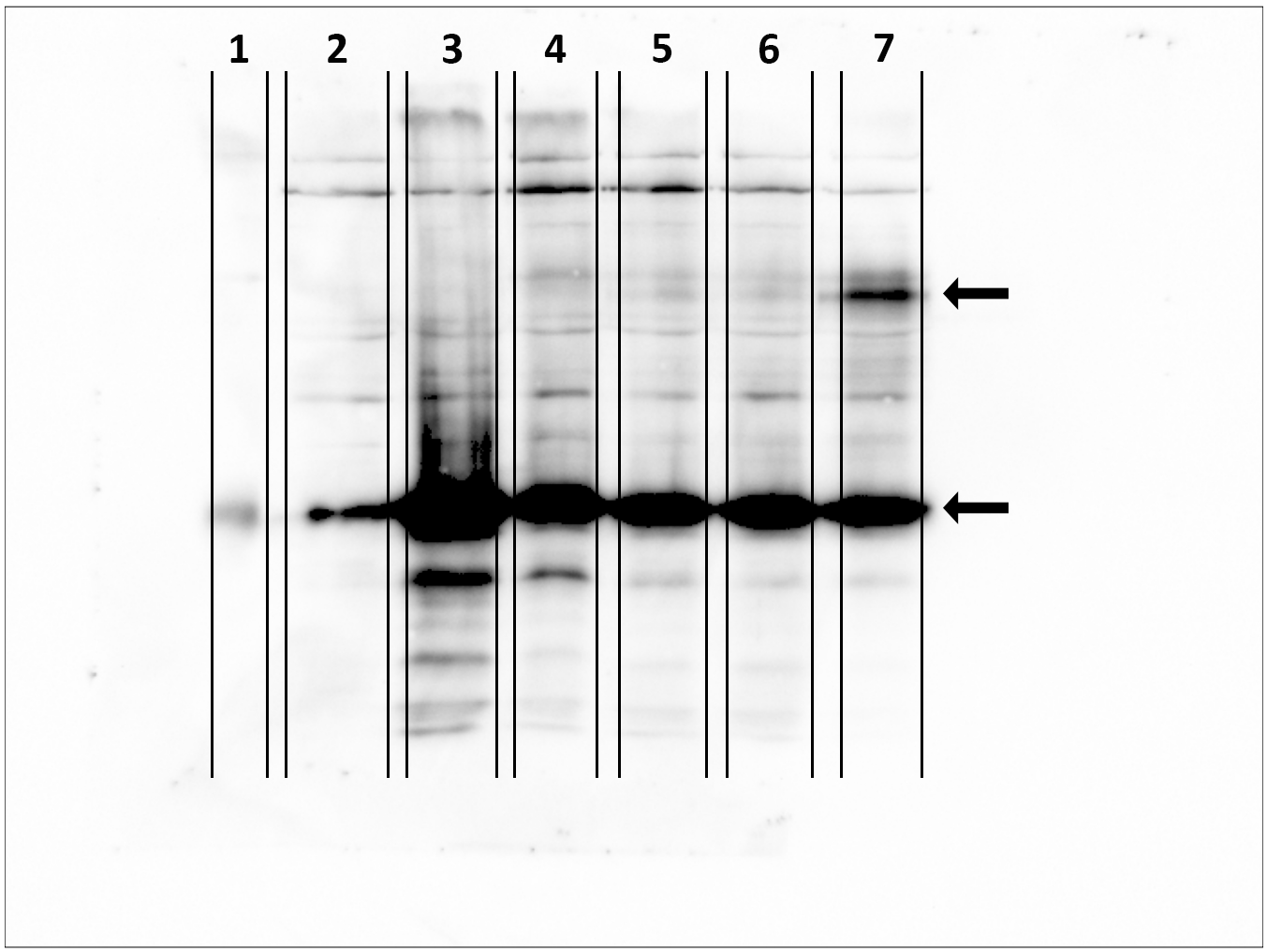


**Figure 5.2. Ngfrb western immunoblot 2** - 1) Marker, 2) uninjected, 3) pT2AL200R150G (Tol2) Empty Vector injection, 4) NgfrbC201-GFP sample group 1 untreated, 5) NgfrbC201-GFP sample group 1 DAPT treated, 6) NgfrbC201-GFP sample group 2 untreated, 7) NgfrbC201-GFP sample group 2 DAPT treated


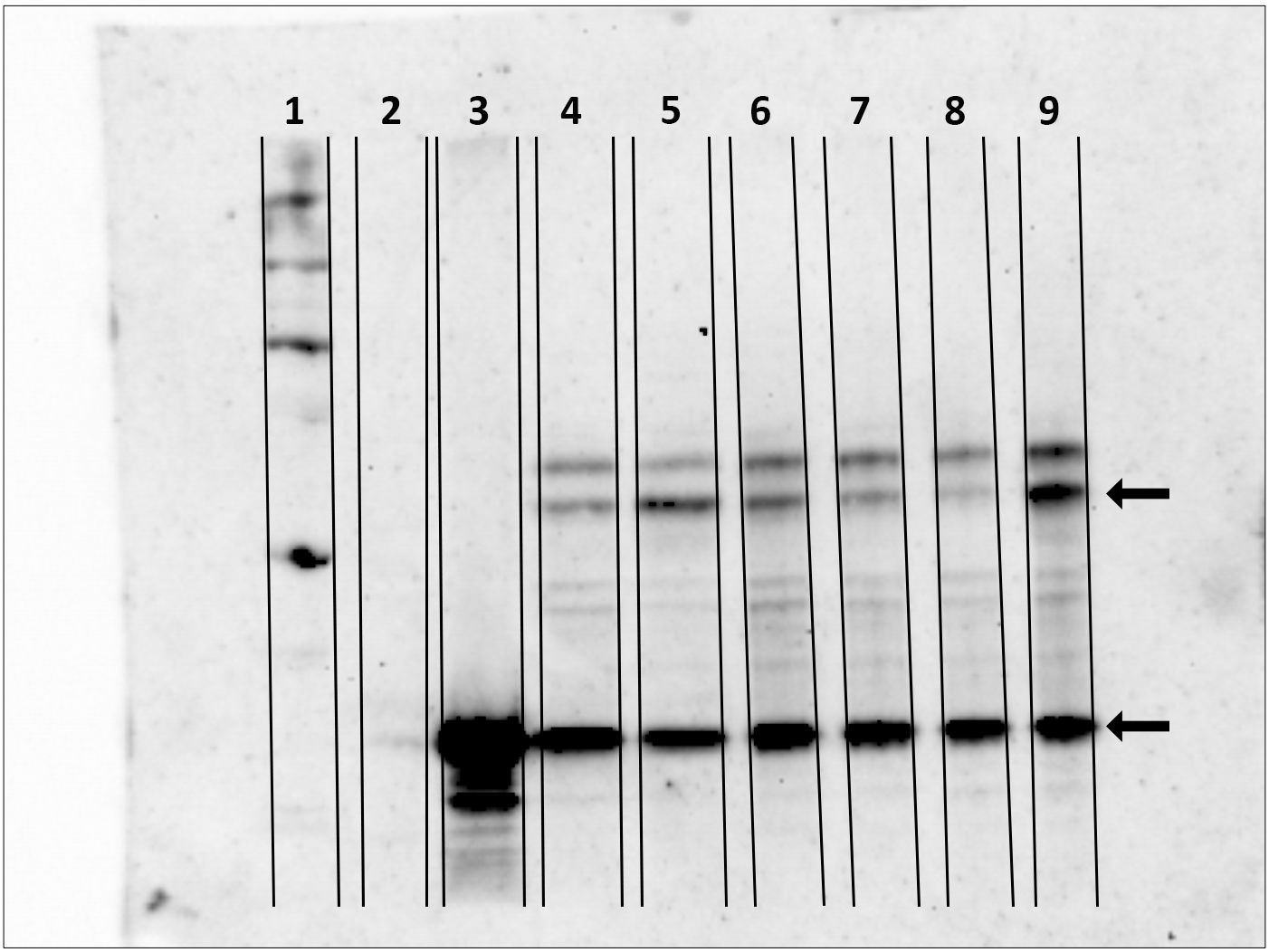


**Figure 5.3. Ngfrb western immunoblot 3** - 1) Marker, 2) uninjected, 3) pT2AL200R150G (Tol2) Empty Vector injection, 4) NgfrbC201-GFP sample group 1 untreated, 5) NgfrbC201-GFP sample group 1 DAPT treated, 6) NgfrbC201-GFP sample group 2 untreated, 7) NgfrbC201-GFP sample group 2 DAPT treated, 8) NgfrbC201-GFP sample group 3 untreated, 9) NgfrbC201-GFP sample group 3 DAPT treated


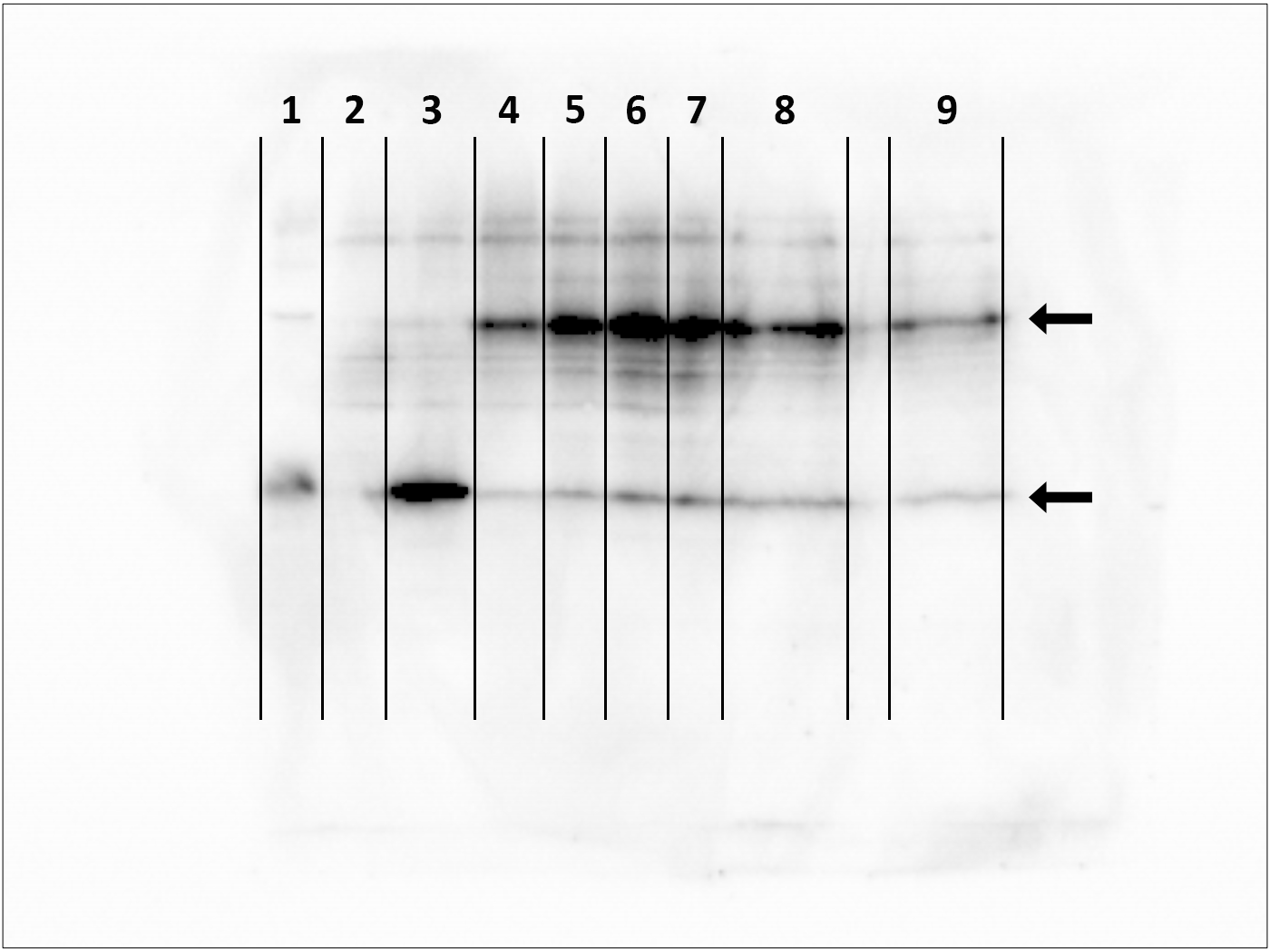


**Figure 5.4. Nradd western immunoblot 1** - 1) Marker, 2) Uninjected, 3) pT2AL200R150G (Tol2) Empty Vector injection, 4) NraddC191-GFP sample group 1 untreated, 5) NraddC191-GFP sample group 1 DAPT treated, 6) NraddC191-GFP sample group 2 untreated, 7) NraddC191-GFP sample group 2 DAPT treated, 8) NraddC191-GFP sample group 3 untreated, 9) NraddC191-GFP sample group 3 DAPT treated


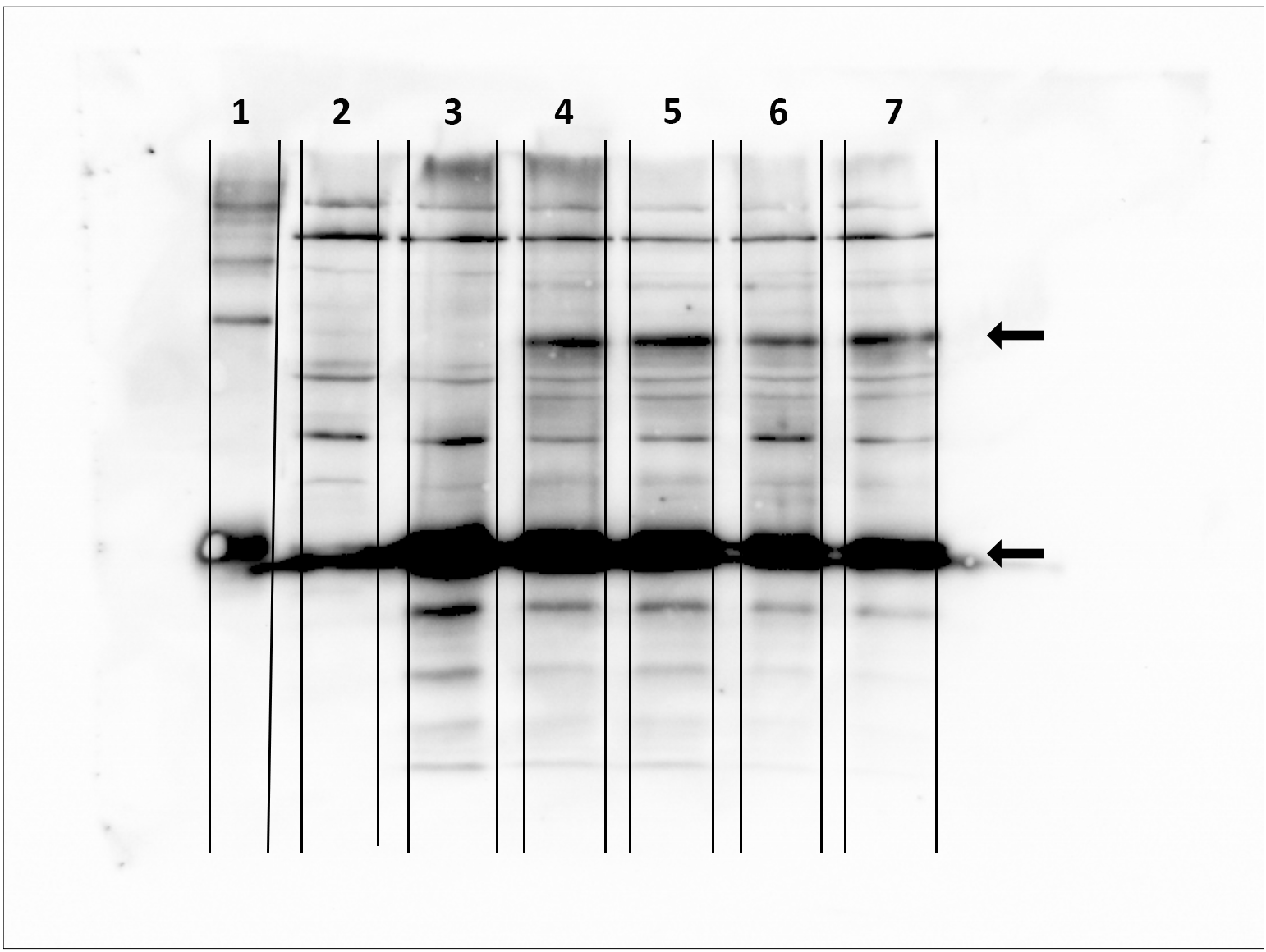


**Figure 5.5. Nradd western immunoblot 2** - 1) Marker, 2) Uninjected, 3) pT2AL200R150G (Tol2) Empty Vector injection, 4) NraddC191-GFP sample group 1 untreated, 5) NraddC191-GFP sample group 1 DAPT treated, 6) NraddC191-GFP sample group 2 untreated, 7) NraddC191-GFP sample group 2 DAPT treated


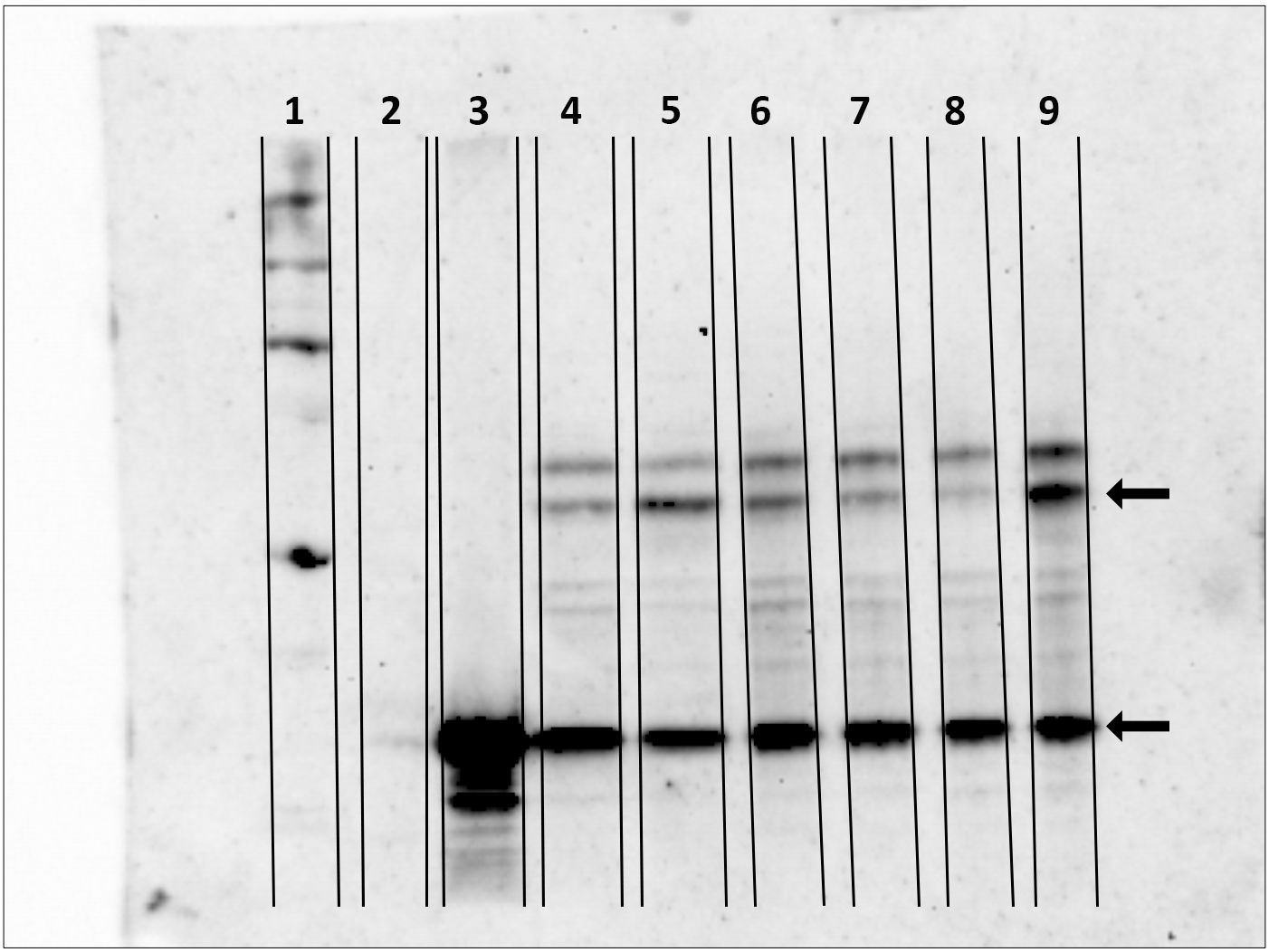


**Figure 5.6. Nradd western immunoblot 3** - 1) Marker, 2) Uninjected, 3) pT2AL200R150G (Tol2) Empty Vector injection, 4) NraddC191-GFP sample group 1 untreated, 5) NraddC191-GFP sample group 1 DAPT treated, 6) NraddC191-GFP sample group 2 untreated, 7) NraddC191-GFP sample group 2 DAPT treated, 8) NraddC191-GFP sample group 3 untreated, 9) NraddC191-GFP sample group 3 DAPT treated


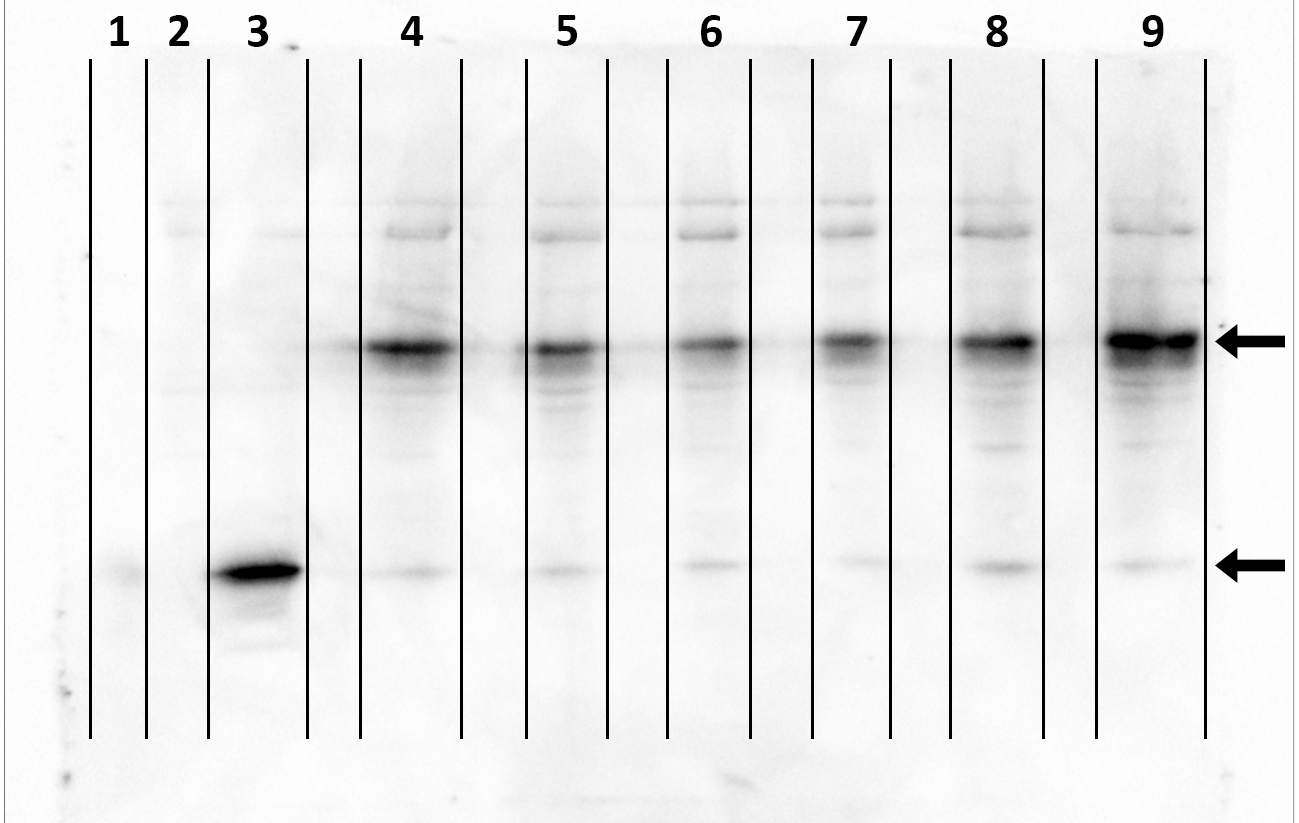


**Figure 5.7. Ngfrb/Nradd “chimera” (A2C) western immunoblot 1** - 1) Marker, 2) Uninjected, 3) pT2AL200R150G (Tol2) Empty Vector injection, 4) A2C-GFP sample group 1 untreated, 5) A2C-GFP sample group 1 DAPT treated, 6) A2C-GFP sample group 2 untreated, 7) A2C-GFP sample group 2 DAPT treated, 8) A2C-GFP sample group 3 untreated, 9) A2C-GFP sample group 3 DAPT treated.


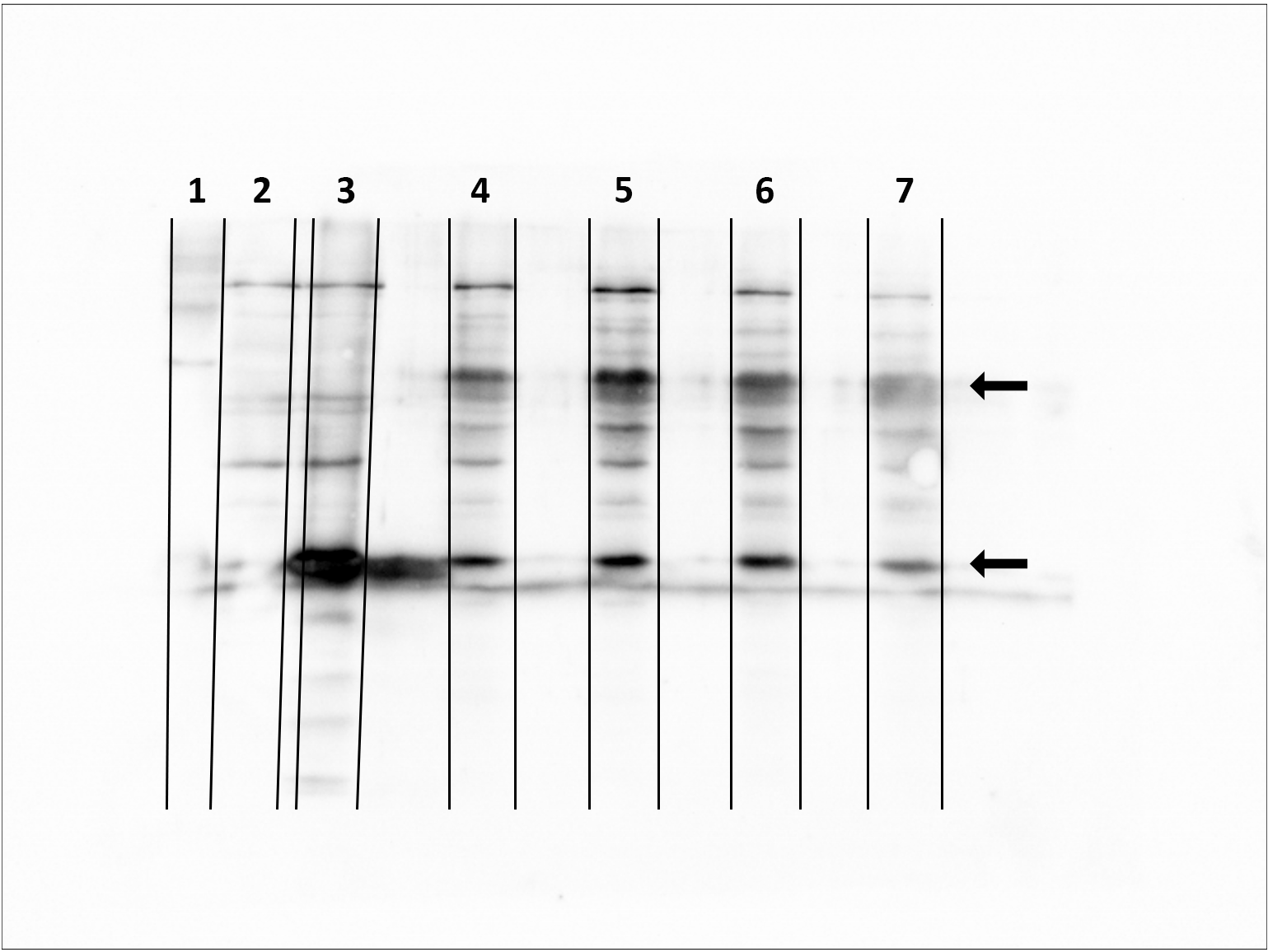


**Figure 5.8. A2C western immunoblot 2** - 1) Marker, 2) Uninjected, 3) pT2AL200R150G (Tol2) Empty Vector injection, 4) A2C-GFP sample group 1 untreated, 5) A2C-GFP sample group 1 DAPT treated, 6) A2C-GFP sample group 2 untreated, 7) A2C-GFP sample group 2 DAPT treated


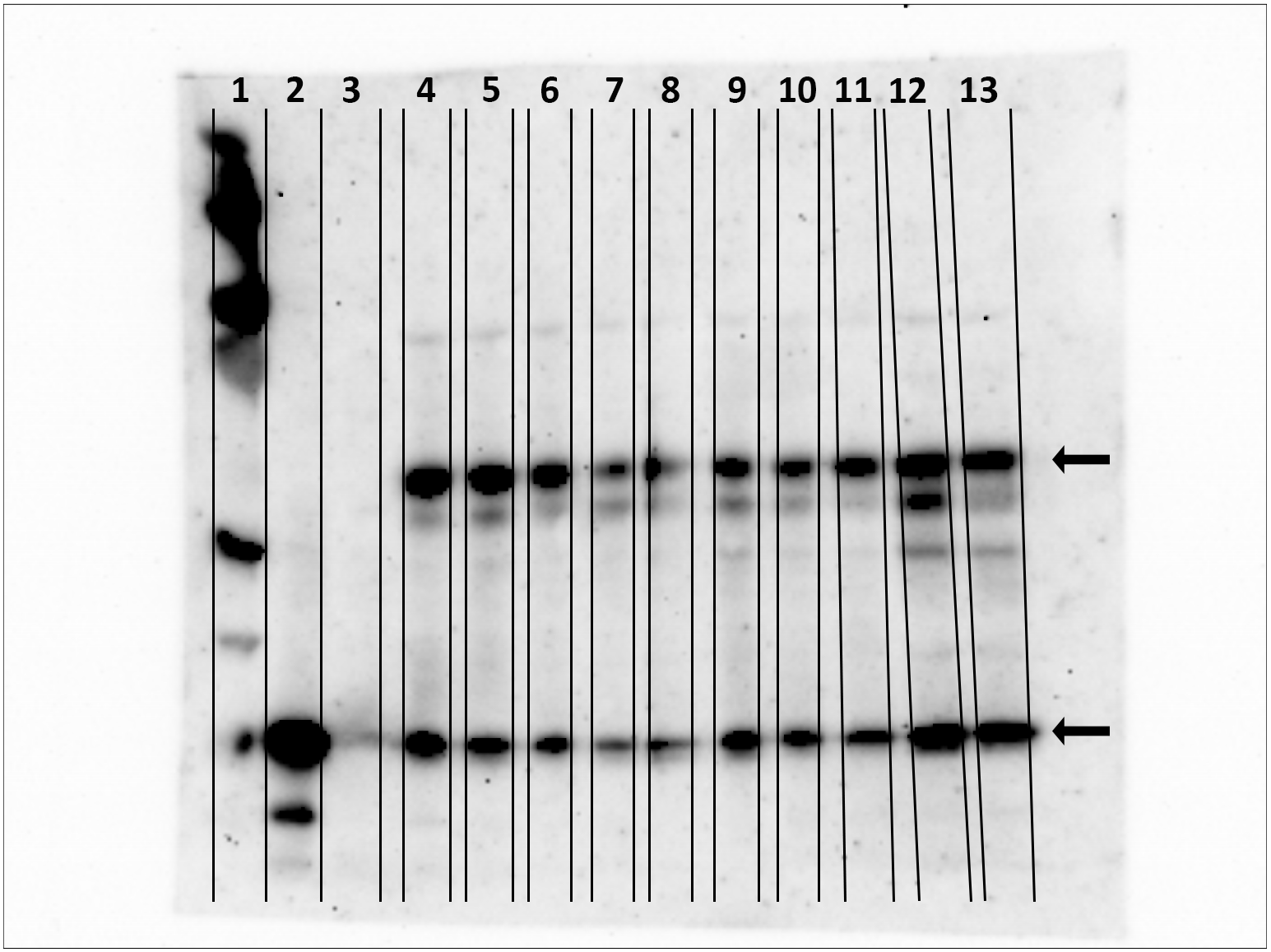


**Figure 5.9. A2C western immunoblot 3** - 1) Marker, 2) Uninjected, 3) pT2AL200R150G (Tol2) Empty Vector injection, 4) A2C-GFP sample group 1 untreated, 5) A2C-GFP sample group 1 DAPT treated, 6) A2C-GFP sample group 2 untreated, 7) A2C-GFP sample group 2 DAPT treated, 8) A2C-GFP sample group 3 untreated, 9) A2C-GFP sample group 3 DAPT treated, 10) A2C-GFP sample group 4 untreated, 11) A2C-GFP sample group 4 DAPT treated, 12) A2C-GFP sample group 5 untreated, 13) A2C-GFP sample group 5 DAPT treated.
